# Brain optimization with additional study time: potential brain differences between high- and low-performance college students

**DOI:** 10.3389/fpsyg.2023.1209881

**Published:** 2023-09-27

**Authors:** Zhiwei Xu, Pengfei Zhang, Mengting Tu, Miao Zhang, Yuanhang Lai

**Affiliations:** School of Business, Hubei University, Wuhan, Hubei Province, China

**Keywords:** high-performance students, low-performance students, EEG, power spectral density, functional connection

## Abstract

This study investigates potential differences in brain function among high-, average-, and low-performance college students using electroencephalography (EEG). We hypothesize that the increased academic engagement of high-performance students will lead to discernible EEG variations due to the brain’s structural plasticity. 61 third-year college students from identical majors were divided into high-performance (*n* = 20), average-performance (*n* = 21), and low-performance (*n* = 20) groups based on their academic achievements. We conducted three EEG experiments: resting state, Sternberg working memory task, and Raven progressive matrix task. Comprehensive analyses of the EEG data from the three experiments focused on power spectral density (PSD) and functional connectivity, with coherence (COH) employed as our primary metric for the latter. The results showed that in all experiments, there were no differences in working memory ability and IQ scores among the groups, and there were no significant differences in the power spectral densities of the delta, theta, alpha1, alpha2, beta, and gamma bands among the groups. Notably, on the Raven test, compared to their high-performing peers, low-performing students showed enhanced functional connectivity in the alpha 1 (8–9 Hz) band that connects the frontal and occipital lobes. We explored three potential explanations for this phenomenon: fatigue, anxiety, and greater cognitive effort required for problem-solving due to inefficient self-regulation and increased susceptibility to distraction. In essence, these insights not only deepen our understanding of the neural basis that anchors academic ability, but also hold promise in guiding interventions that address students’ diverse academic needs.

## 1. Introduction

Students’ academic performance is influenced by a multitude of factors, and extensive literature exists on this topic (Tracey et al., 2012; Kurup and Subramanyam, 2014; Burger and Naude, 2020; Vrapi et al., 2022). However, when it comes to the academic performance of college students, there is a consensus: high-performing students tend to dedicate significant time and effort to their studies, while low-performing students often allocate less study time, leading to higher rates of academic failure (Montes Iturrizaga, 2012; Cerna and Pavliushchenko, 2015). Previous research has linked low-performance students to a lack of interest in their majors, leading to inadequate high-quality study time and subsequent academic failure (Wang and Eccles, 2013). Other studies have found that high-performance students tend to set high goals for themselves, and increased learning motivation is stimulated by high learning goals, thus resulting in more time invested in learning. At the same time, low-performance students have lower academic standards for themselves, while learning motivation is not high. Consequently, they invest less time in learning (Martínez-Monteagudo et al., 2018). This trend has been consistently observed in educational research, establishing that high-performing students invest more time in studying at universities, while low-performing students allocate less time to academics (Díaz-Mora et al., 2016).

Learning is a highly mentally consuming activity, especially during high-intensity learning, and the demand of the brain for blood is six times higher than usual (Hampshire et al., 2019), At the same time, learning is also a high-training activity for the brain. There is a large amount of evidence showing that hard study can effectively train the brain. As we continue to learn, think, and practice in a specific field, the synaptic plasticity of the brain in this aspect will be significantly enhanced (Taya et al., 2015; Weicker et al., 2016). This is because the brain has structural plasticity, in which it usually learns to modify the connections between synapses to acquire new brain structures and new behavioral capabilities (Poldrack, 2000; Kolb et al., 2003). Consequently, the divergent college experiences of high-performance and low-performance students create contrasting brain environments, prompting the question of whether these two groups exhibit distinct brain characteristics, detectable through EEG features.

Therefore, divergent college experiences (self-disciplined vs. indulgent approaches) have given rise to two distinct brain groups exposed to contrasting environments (consistent vs. minimal stimulation). Therefore, do the brains of these two groups exhibit differences, and can these disparities be discerned in electroencephalogram (EEG) features? It can be postulated that students from the same major and university possess no significant differences in brain function upon entering university. Admissions criteria for the same major at the same university tend to be consistent (e.g., equivalent college entrance examination scores), which, to a degree, filters for IQ and other brain functions related to learning ability (e.g., working memory). The variation in IQ among students within the same major at the same university is anticipated to be negligible, a notion supported by research dating back to the 1950s (Plant and Richardson, 1958). In our study, we selected third-year students from the same major at a highly competitive university with exceptionally high college entrance examination scores as participants. It can be assumed that among this cohort of third-year students, no significant differences in brain functions associated with academic abilities existed between high-performance, average, and low-performance students upon their admission to the university. Otherwise, they would not have been accepted into the university and major with identical admissions criteria. It is worth noting that the highly selective admission criteria of this university, predicated on exceedingly high college entrance examination scores, can be regarded as a form of cognitive and brain function ability selection. Thus, if the brains of high-performance and low-performance students exhibit differences in EEG after 3 years of college life, the primary explanation is that the brains of high-performance students have undergone comprehensive training due to rigorous studying, while low-performance students have experienced limited study and insufficient brain training. This is the central theme that our study endeavors to investigate and substantiate. We acknowledge that there are other factors that can influence brain function ability, such as alcoholism (Correas et al., 2015; Kim et al., 2020), medication use (Coullaut-Valera et al., 2014), sleep disorders (Peter-Derex et al., 2021), and neurological or brain disorders (Alturki et al., 2020). To mitigate the impact of these confounding factors, rigorous subject screening was conducted to exclude individuals who may be affected. By employing this stringent selection process, we aimed to minimize their potential influence and ensure the integrity of our study outcomes.

## 2. Theoretical basis and research hypothesis

Brains that have undergone training are anticipated to exhibit significant differences compared to untrained brains, and these substantial disparities can be discerned in resting-state EEGs, as corroborated by numerous studies (Vernon et al., 2003; Angelakis et al., 2007; Karbach and Schubert, 2013; Rose et al., 2015). For instance, a recent investigation conducted at Stanford University demonstrated an increased involvement of the hippocampal learning and memory system in the brains of college students exhibiting positive academic attitudes and diligent study habits in mathematics (Chen L. et al., 2018). Resting-state EEGs of long-term meditators differed markedly from those of control group participants, particularly at the lateral frontoparietal lobe electrodes. Long-term meditators exhibited an elevated ratio of γ-band activity (25–42 Hz) to slow oscillation activity (4–13 Hz; Lutz et al., 2004). After a three-month period of intensive meditation training, patients with attention deficit disorder exhibited enhanced brain function by increasing the phase consistency of θ-band oscillatory neural responses in the forebrain and reducing response time variability (Lutz et al., 2009).

Even in aging and deteriorating cerebral cortices, the resting EEG of trained brains exhibits marked distinctions compared to their untrained counterparts. Following training, cognitive control capacity is enhanced among older adults aged 60–85 years, accompanied by improvements in sustained attention and working memory. These individuals demonstrate a decrease in multitasking costs relative to active and no-contact control groups, surpassing the performance of untrained 20-year-old subjects, with sustained gains for a duration of 6 months. Additionally, age-related impairments in neural markers of cognitive control, as assessed via EEG, were alleviated by multitasking training (e.g., augmented midline frontal theta power and frontal-posterior theta coherence; Anguera et al., 2013).

The aforementioned investigation substantiates the discernable disparities between trained and untrained brains concerning power spectra and resting functional connectivity. On one hand, high-performance students have devoted considerable time to academic pursuits at the university level, receiving extensive cerebral stimulation over multiple years. Conversely, the brains of low-performing students have not been subjected to equivalent cognitive training. Are there significant variations in the resting EEG patterns between these two cohorts? Building upon the research outlined above, we formulated our initial hypothesis:

*H1*a: High-performing and low-performing third-year students majoring in the same course at the same university will display differences in their whole brain power spectrum of resting-state EEG.

*H1*b: High-performing and low-performing third-year students majoring in the same course at the same university will exhibit differences in the functional connectivity (coherence) of their resting-state EEG.

Trained cerebral cortices exhibit substantial dissimilarities from untrained ones, extending beyond resting states to encompass task states as well. This is most prominently demonstrated in working memory tasks, where functional training has been shown to enhance the brain’s working memory capacity (Weicker et al., 2016). Theta and alpha bands in the frontal region have traditionally been considered the two frequency bands most intimately associated with working memory (Klimesch, 1999a; Wei and Zhou, 2020), Nevertheless, recent findings indicate a strong connection between the delta band and working memory (Akturk et al., 2022). Another EEG metric reflecting working memory alterations is the brain network index. In working memory tasks, the functional connectivity of trained brains’ neuronal networks within the frontal–parietal and occipital regions undergoes change (Anguera et al., 2013; Langer et al., 2013). A wealth of evidence supports the notion that working memory serves as a predictor of academic achievement (Swanson and Alloway, 2012). Studies have elucidated the relationship between fluid intelligence and complex learning (Wang et al., 2013), and indeed, working memory measurements have consistently demonstrated a superior ability to forecast academic aptitude compared to intelligence metrics (Alloway et al., 2013). Titz and Karbach (2014) conducted a comprehensive examination of research spanning two decades on working memory training and its influence on academic performance. Their analysis unveiled constrained yet consistent evidence endorsing the positive impact of process-based complex working memory training on academic skills, particularly in the domain of reading comprehension. These advantages were observed among children presenting cognitive and academic deficits, as well as in cognitively healthy students (Titz and Karbach, 2014). Based on this evidence, we formulate the following hypotheses:

*H2*a: High-performing third-year students from the same university majoring in the same course and performing the same working memory task will exhibit superior working memory compared to their low-performing counterparts.

*H2*b: The brain power spectrum of EEG will differ between high-performing and low-performing third-year students from the same university majoring in the same course and performing the same working memory task.

*H2*c: The brain functional connectivity (coherence) of EEG will differ between high-performing and low-performing students from the same university majoring in the same course and performing the same working memory task.

In addition to working memory tasks, trained cerebral cortices display marked disparities in EEG parameters relative to untrained counterparts during tasks assessing global cognitive function. Numerous studies have demonstrated that, despite possessing equivalent IQ scores, high- and low-performing students exhibit significant neural differences in EEG tasks related to general brain function. For instance, Staudt and Neubauer assessed the cerebral activity of adolescents with average IQ scores who were classified as academic achievers and underachievers. They discovered that high-performing students exhibited greater posterior brain activation than their underperforming peers during Posner letter-matching tasks (Staudt and Neubauer, 2006). Further research has indicated that, among students with uniformly high IQ scores, underachievers displayed reduced prefrontal activation compared to high achievers when engaged in creative tasks (Bergner and Neubauer, 2011). However, the participants in the aforementioned investigations were not drawn from the same university or academic discipline. Consequently, we sought to determine whether significant differences in EEG parameters exist between high- and low-performing students within the same university and major with respect to comprehensive cognitive ability tasks. This inquiry prompted the formulation of our third hypothesis:

*H3*a: There will be no significant IQ difference between high-performing and low-performing third-year students in the same university and major.

*H3*b: The brain power spectrum of EEG will differ between high-performing and low-performing third-year students from the same university majoring in the same course and performing the same comprehensive ability task of brains.

*H3*c: Concerning the comprehensive ability task of brains, there will be differences in the function connection (coherence) of EEG between high- and low-performance third-year students.

## 3. Materials and methods

### 3.1. Subjects

A total of 76 third-year students majoring in Business Administration from a reputable business school were initially recruited for this study, all of whom entered their college course with comparable college entrance examination scores. Prior to the experiment, left-handed students, students with neurological disorders, students with alcoholism or sleep disorders, and those who had recently consumed psychotropic drugs were excluded. Among the remaining students, 69 were selected based on the weighted scores of their previous five semesters. The top 15% were designated as “high-performance students,” the bottom 15% as “low-performance students,” and those within the 45–55% score gradient were assigned to the “normal student group,” which served as the control group. This classification was established by the teaching office of the participants’ university and solely relied on academic performance within the students’ major. After preprocessing EEG data, an additional 8 participants were excluded because their valid data segments did not reach 80%. In accordance with common practices in neuroscience research and considering previous literature and resource constraints, a sample size of approximately 60–70 participants was deemed appropriate for EEG studies (Rogasch et al., 2015; Harris et al., 2020; Pavlov et al., 2021; Shadli et al., 2021).

The final sample consisted of 61 subjects with an average age of 20.4 ± 0.87 years, including 36 females. All participants provided informed consent prior to their involvement in the study. The sample included 20 high-performance students (12 females), 21 normal students (13 females), and 20 low-performance students (11 females) (Table 1). To account for the average weekly study time of all students, excluding university class time, data were obtained not from the subjects themselves but from two roommates of each participant through interviews with their counselors, thereby mitigating potential exaggeration of self-reported study time. Additionally, information regarding the number of failed courses for each participant over the past five semesters and their absenteeism records for the current semester were collected from the university’s academic affairs office. These supplementary data provided a comprehensive understanding of the academic performance and behavior of the study participants.

**Table 1:** Demographic characteristics of study participants.

Student type	Average age	Age standard deviation	Female count	Male count
High-performance students	20.00	0.79	12	8
Normal students	20.62	0.97	13	8
Low-performance students	20.45	0.89	11	9

### 3.2. Materials and procedures

**Figure 1:**
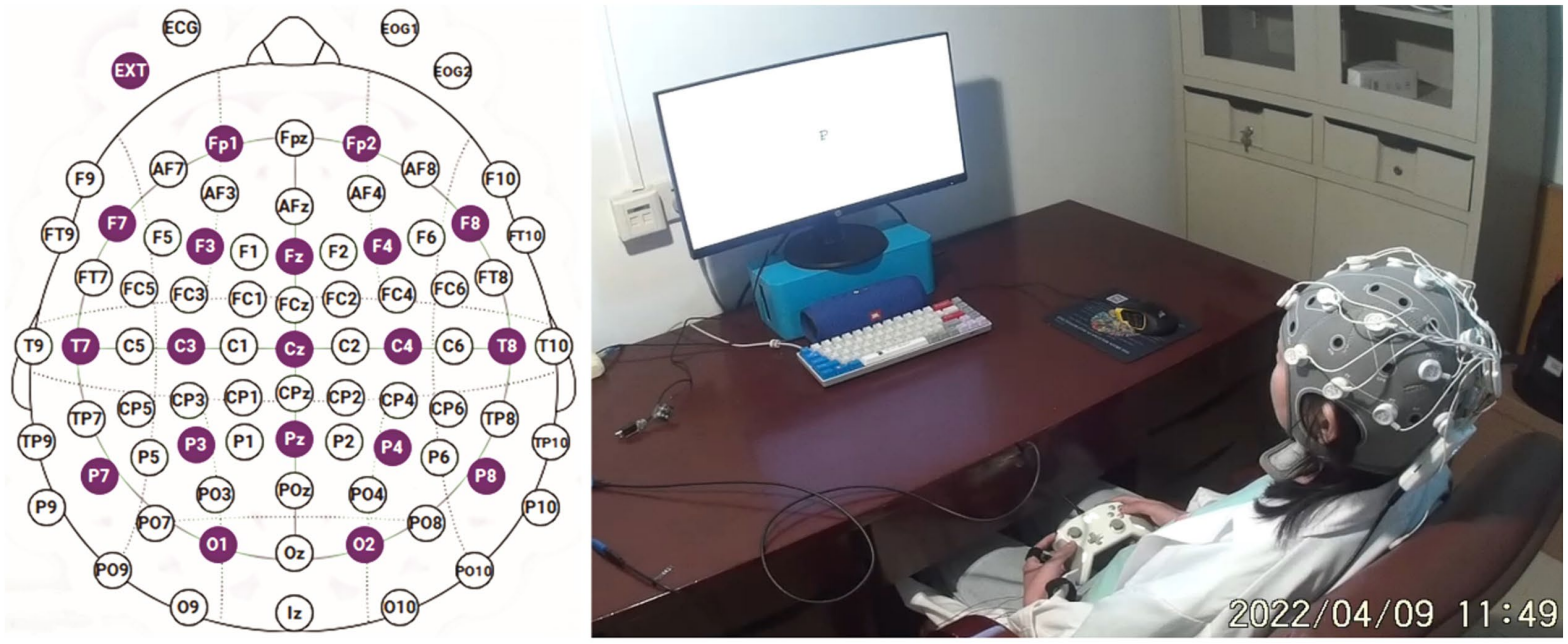
The electrode position map and on-site experimental photo.

An Enobio20 EEG system (NeuroElec) was employed for the experiments, with recordings adhering to the international 10–20 system’s 20-conductive polar cap. Electrode impedances were set at a 10 kOhm threshold, and electrode positions are depicted in Figure 1. The right ear clip electrode served as the reference electrode, and the equipment’s sampling rate was 500 Hz. Experiments were conducted in a laboratory under DC lamp illumination, with subjects donning EEG helmets, sitting on a cushioned sofa, and facing a 23-inch monitor with a 60 Hz refresh rate. Feedback and output were acquired through an Xbox controller on the participants’ thighs. Prior to the experiment, subjects adjusted their seating for maximum comfort and relaxation, with only the right thumb controlling the joystick. E-prime 3.0 was used for loading all experimental materials.

#### 3.2.1. Experiment 1: resting electroencephalography

Before commencing the experiment, participants were briefed on precautions for the EEG experiment, including avoiding movement, talking, frequent blinking, and significant facial expressions. Equipped with this knowledge and the EEG apparatus, subjects’ resting EEG data were collected for 5 min while they focused on a central “+” symbol displayed on the screen and remained relaxed. In the realm of neuroscientific investigations, particularly for scenarios necessitating swift evaluations or preliminary screenings, a 3–5 min recording of resting-state EEG is deemed sufficient to capture the brain’s fundamental activities and potential anomalies (Pfurtscheller et al., 2012). Such a duration not only captures essential neural dynamics but also ensures feasibility and efficiency in data acquisition. This perspective aligns with recent findings that have utilized short-duration EEG recordings to elucidate brain dynamics and their clinical implications (Niedermeyer and Da Silva, 2005). In line with this understanding, the data duration adopted in our study, specifically a 5-min resting-state EEG recording, is both academically acceptable and methodologically sound.

#### 3.2.2. Experiment 2: Sternberg working memory (STB) task

The STB paradigm materials were sourced from the Psychology Software Tools official website (PST experiment number 3012) and implemented using E-prime3.0 (PST Admin, 2022). This classic experimental scheme is widely employed in working memory research to assess subjects’ storage and retrieval of items in short-term memory (Raghavachari et al., 2001; Brookes et al., 2011). First, a series of letters (one letter/s; eight letters in total) were shown to the subjects. Among the displayed letters, the participants had to memorize the black letters. At the same time, the participants were not required to remember green letters. The ratio of black to green letters was 7:1 (the default setting in the STB experimental paradigm).

**Figure 2:**
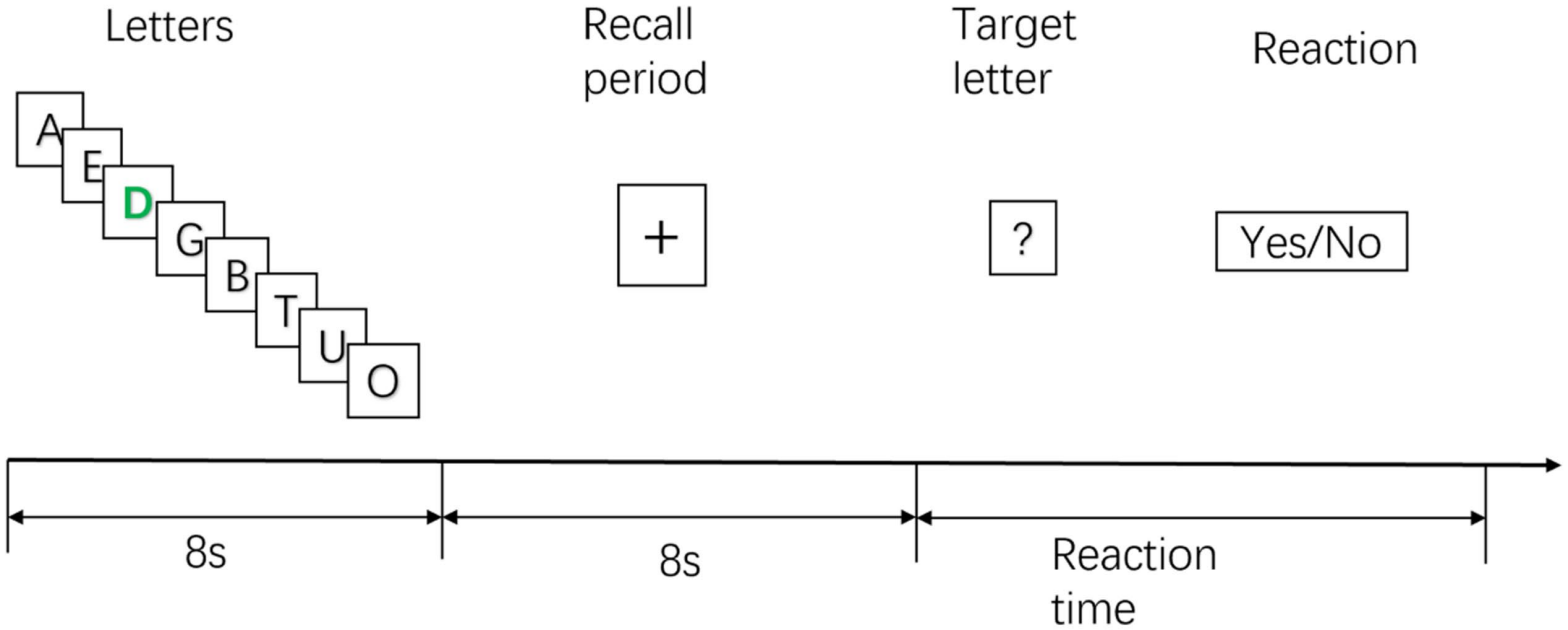
Sternberg working memory (STB) experimental diagram.

Subjects are required to memorize these letters in short-term memory. After all the letters were displayed, the subjects were required to memorize them, using their short-term memory, and keep them for 8 s. Then, they were shown seven target letters. Subjects were required to judge whether the target letters had appeared in the previous memorization process. If the target letters matched the previously displayed letters, the subjects quickly pressed the A button on the handle. The participant quickly pressed the B button on the handle if the target letter was new. There was a 5-s pause after all the subjects had finished their answers. The next experimental cycle would then begin. This study was conducted for two experimental cycles of 5 min. The entire experimental process is shown in Figure 2.

#### 3.2.3. Experiment 3: comprehensive brain ability task

**Figure 3:**
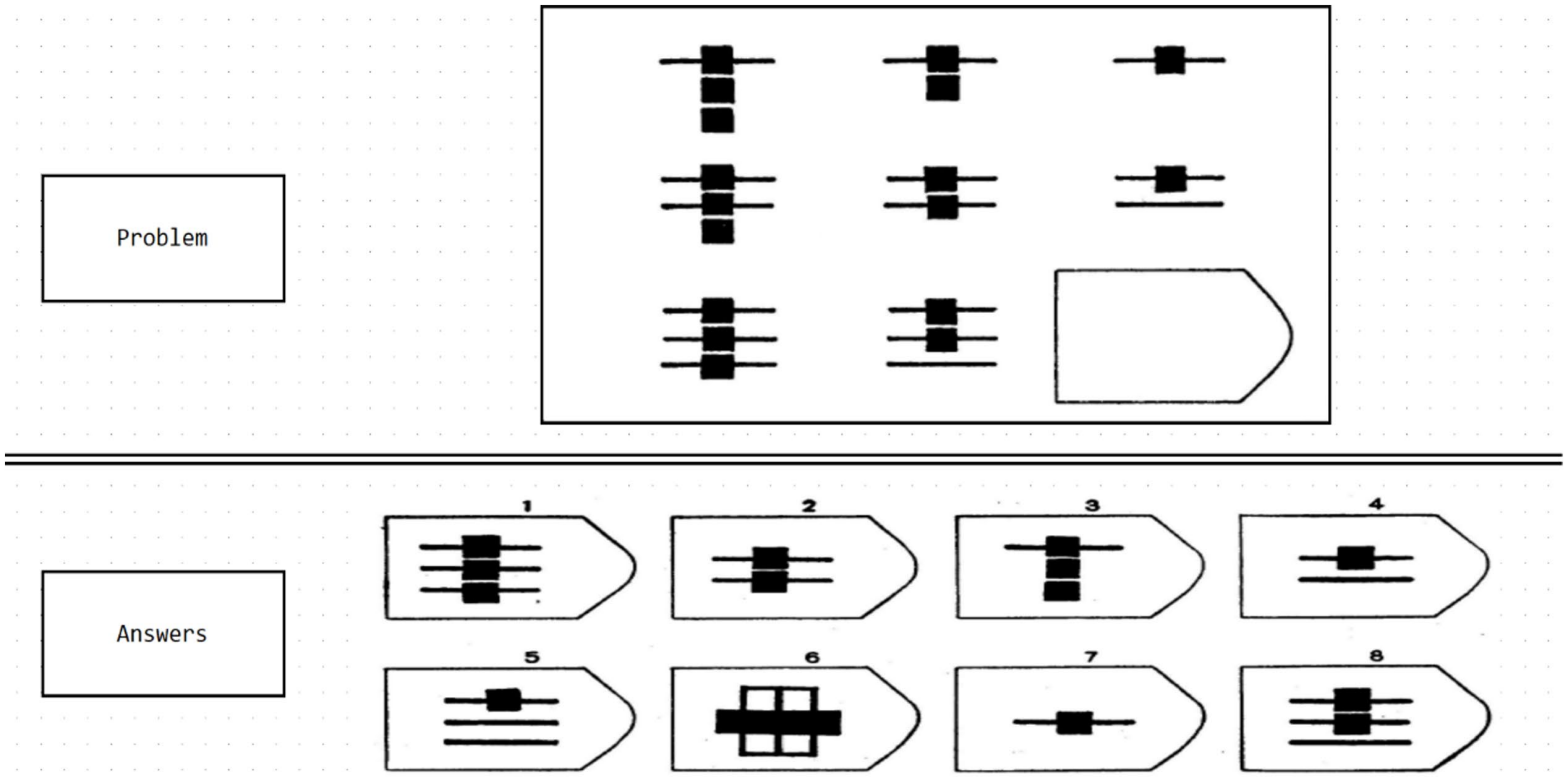
Diagram of the Raven test experimental set used in this study. Source: Raven progressive matrices from the PST website (PST experiment number 34568).

**Figure 4:**
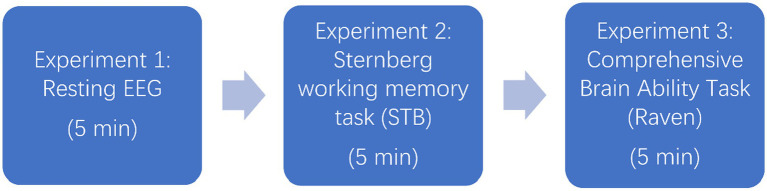
Experimental flowchart.

Experiment 3 utilized materials from the Raven Progressive Matrices on the Psychology Software Tools website (PST experiment number 34568; PST Admin, 2023). In this test, the comprehensive ability of the brain of the subject is examined through a series of regular geometric figures (Figure 3). This test evaluates subjects’ comprehensive brain abilities through a series of regular geometric figures (Figure 4). The Raven test, which assesses various psychological resources such as task-related knowledge, working memory, attention, and decision-making (Friedman et al., 2019), is an appropriate measure of comprehensive brain ability, boasting high reliability, validity, and independence from cultural constraints (Raven, 2000). The Raven test, in combination with EEG, has been widely endorsed for evaluating comprehensive brain ability (Schabus et al., 2006; Amin et al., 2015; Friedman et al., 2019). Subjects completed the 40-min experiment using an Xbox controller to select correct geometric figures. Due to the impracticality of analyzing the full 40-min EEG data from each participant—resulting in several 100 gigabytes of data and exceeding our computer system’s capacity—only the initial 5 min of EEG data from each participant were analyzed, which is sufficient for most EEG analyses.

### 3.3. EEG data analysis

#### 3.3.1. Electroencephalography raw data preprocessing

EEGLAB (version 2021), operating on MATLAB R2021b, was employed for data preprocessing (Delorme and Makeig, 2004). Initially, band-pass (1 and 100 Hz) and notch filters (48–52 Hz) were applied. Data were segmented into 2-s intervals, and the ICA analysis was conducted using the ADJUST1.1.1 plug-in to eliminate artifacts such as blinking, eye movement, electromyography, and Electrocardiogram. Data segments exceeding ±100 μV were removed. Post-preprocessing, approximately 80% of each subject’s valid segments were retained.

#### 3.3.2. Frequency domain analysis

Power spectral density (PSD) reveals the energy distribution of EEG signals across various frequency bands and electrodes. This study employed a fast Fourier transform (FFT) to conduct PSD analysis on three groups of subjects participating in three experiments. The analyzed frequency bands included delta (1–4 Hz), theta (4–8 Hz), alpha1 (8–9 Hz), alpha2 (10–13 Hz), beta (13–30 Hz), and gamma (30–80 Hz). The PSD is computed using the Fourier Transform and is defined as follows:


(1) $$PSD\left(f\right)=\frac{1}{T}{\left|F\left\{x\left(t\right)\right\}\right|}^{2}$$

Where:

$PSD\left(f\right)$ represents the Power Spectral Density at frequency f , measured in $\mu V^{2}/\mathrm{Hz}\text{.}$

T is the total duration of the EEG signal in time.

$x\left(t\right)$ is the EEG signal in the time domain.

$F\left\{x\left(t\right)\right\}$ denotes the Fourier Transform of $x\left(t\right)$

This formula provides a quantitative measure of the EEG signal’s power distribution across various frequencies. The unit of the PSD is $\mu V^{2}/\mathrm{Hz}$ , which is in accordance with the standard conventions for EEG analysis. A MATLAB2021-based programming script was utilized to facilitate the aforementioned process.

#### 3.3.3. Functional connection analysis

The linear relationship between two signals at a particular band or frequency point is measured with coherence (COH; Niso et al., 2013). Suppose that $X\left(t\right)$ and $Y\left(t\right)$ represented the EEG signals from different electrodes (or brain regions) X and Y , respectively. First, the time domain signal is converted to the frequency domain using the constant frequency domain conversion method of the fast Fourier transform. For each frequency f , its power spectral densities $S_{xx}\left(f\right)$ and $S_{yy}\left(f\right)$ and their cross-power spectral densities $S_{xy}\left(f\right)$ were calculated. Accordingly, the coherency function $K_{xy}\left(f\right)$ is computed coherently using the following formula:


(2) $$K_{xy}\left(f\right)=\frac{S_{xy}\left(f\right)}{\sqrt{S_{xx}\left(f\right)S_{yy}\left(f\right)}}$$

Then the following formula is used to calculate the coherence value at frequency f :


(3) $$COH_{xy}\left(f\right)={\left|K_{xy}\left(f\right)\right|}^{2}$$

The coherence index ranged from 0 to 1. $COH_{xy}\left(f\right)=0$ means that there is no linear dependence between $X\left(t\right)$ and $Y\left(t\right)$ at frequency f . The larger the coherence value, the stronger the statistical dependence between the two signals, and vice versa. Coherence (COH) is a widely used measure in EEG data analysis to assess the functional connectivity between two regions of the brain. Essentially, it quantifies the consistency of the phase difference between signals from two separate EEG channels. A high coherence value indicates that the two regions (or channels) are functionally connected or are working together, while a low coherence suggests little to no connection. In this study, a programming script based on MATLAB2021 was used to realize the above process.

#### 3.3.4. Multiple comparison correction

The first kind of error (false positive) in statistics is controlled by the significance level a , if m independent comparisons are made, especially when the value of m is large, even if there is no difference between the samples under the two conditions, the probability of being detected with one or more false positives is considerable. Still, it cannot be guaranteed to be below the significance level a . In this study, the false discovery rate (FDR) method is used to correct multiple comparisons. The FDR was the most commonly used tool for multiple comparisons in the current EEG analysis. This method ensured weak control of the overall first-class error rate. The overall first-class error rate can only be effectively controlled if the original assumptions are accurate (Wu et al., 2011). We employed the “*mafdr*” function in MATLAB 2021 for implementing multiple comparison correction. The value of p for false discovery rate (FDR) was set at a threshold of 0.05.

## 4. Results

### 4.1. Weekly study time and academic performance

Table 2 presents the ANOVA analysis results for academic performance, weekly study time, number of absences, and number of failed courses among the three subject groups. The statistical analysis was conducted using SPSS 27, employing one-way ANOVA with dependent variables “Average study time per week,” “Weighted average grade score,” “Number of failed courses,” and “Number of absenteeism,” with the factor of “Academic performance group.” Statistical tests such as the Welch test, Tukey’s *post-hoc* multiple comparison test, and the Games-Howell test were applied when equal variances were not assumed.

**Table 2:** Descriptive statistics and ANOVA results of the “average weekly study time,” “academic achievements,” “Number of failed courses” and “Number of absenteeism” of the three groups in this study.

Descriptives									
		N	Mean	Std. deviation	Std. error	95% confidence interval for mean		Minimum	Maximum
						Lower bound	Upper bound		
Average study time per week	High performance	20	36.15	4.91	1.36	33.18	39.12	30.00	46.00
	Normal students	21	20.08	3.96	1.14	17.56	22.60	10.00	25.00
	Low performance	20	1.54	1.80	0.50	0.45	2.62	0.00	6.00
	Total	61	19.24	14.98	2.43	14.31	24.16	0.00	46.00
Weighted average grade score	High performance	20	85.13	0.97	0.27	84.54	85.71	83.20	86.31
	Normal students	21	77.16	4.26	1.23	74.45	79.87	70.00	80.94
	Low performance	20	55.21	7.11	1.97	50.91	59.50	41.02	65.07
	Total	61	72.38	13.79	2.24	67.84	76.91	41.02	86.31
Number of failed courses	High performance	20	0.00	0.00	0.00	0.00	0.00	0.00	0.00
	Normal students	21	0.50	0.67	0.19	0.07	0.93	0.00	2.00
	Low performance	20	5.00	3.08	0.85	3.14	6.86	3.00	15.00
	Total	61	1.87	2.91	0.47	0.91	2.83	0.00	15.00
Number of absenteeism	High performance	20	0.08	0.28	0.08	−0.09	0.24	0.00	1.00
	Normal students	21	0.67	0.89	0.26	0.10	1.23	0.00	2.00
	Low performance	20	10.31	4.85	1.35	7.37	13.24	0.00	19.00
	Total	61	3.76	5.55	0.90	1.94	5.59	0.00	19.00

ANOVA						
		Sum of squares	df	Mean square	F	Sig.
Average study time per week	Between groups	7801.03	2.00	3900.51	272.31	0.000
	Within groups	501.34	58.00	14.32		
	Total	8302.37	60.00			
Weighted average grade score	Between groups	6219.17	2.00	3109.59	133.21	0.000
	Within groups	817.01	58.00	23.34		
	Total	7036.19	60.00			
Number of failed courses	Between groups	195.34	2.00	97.67	28.73	0.000
	Within groups	119.00	58.00	3.40		
	Total	314.34	60.00			
Number of absenteeism	Between groups	848.51	2.00	424.25	50.79	0.000
	Within groups	292.36	58.00	8.35		
	Total	1140.87	60.00			

Table 2 revealed that students with lower academic performance dedicated less time to studying each week, while those with higher performance demonstrated greater self-discipline, investing more time in their studies. Students with average performance exhibited intermediate weekly study times between these two groups. Additionally, the low-performance group experienced higher absenteeism rates and more failed courses compared to the high-performance group, who maintained perfect attendance and had no failed courses. The normal-performance group fell between the high- and low-performance groups regarding absenteeism and failed courses. The stark contrast between the low-performance group’s high absenteeism and minimal study time and the high-performance group’s exemplary self-discipline and attendance underscores the significance of self-regulation and dedication in academic achievement.

### 4.2. Resting-state spectrum analysis

**Figure 5:**
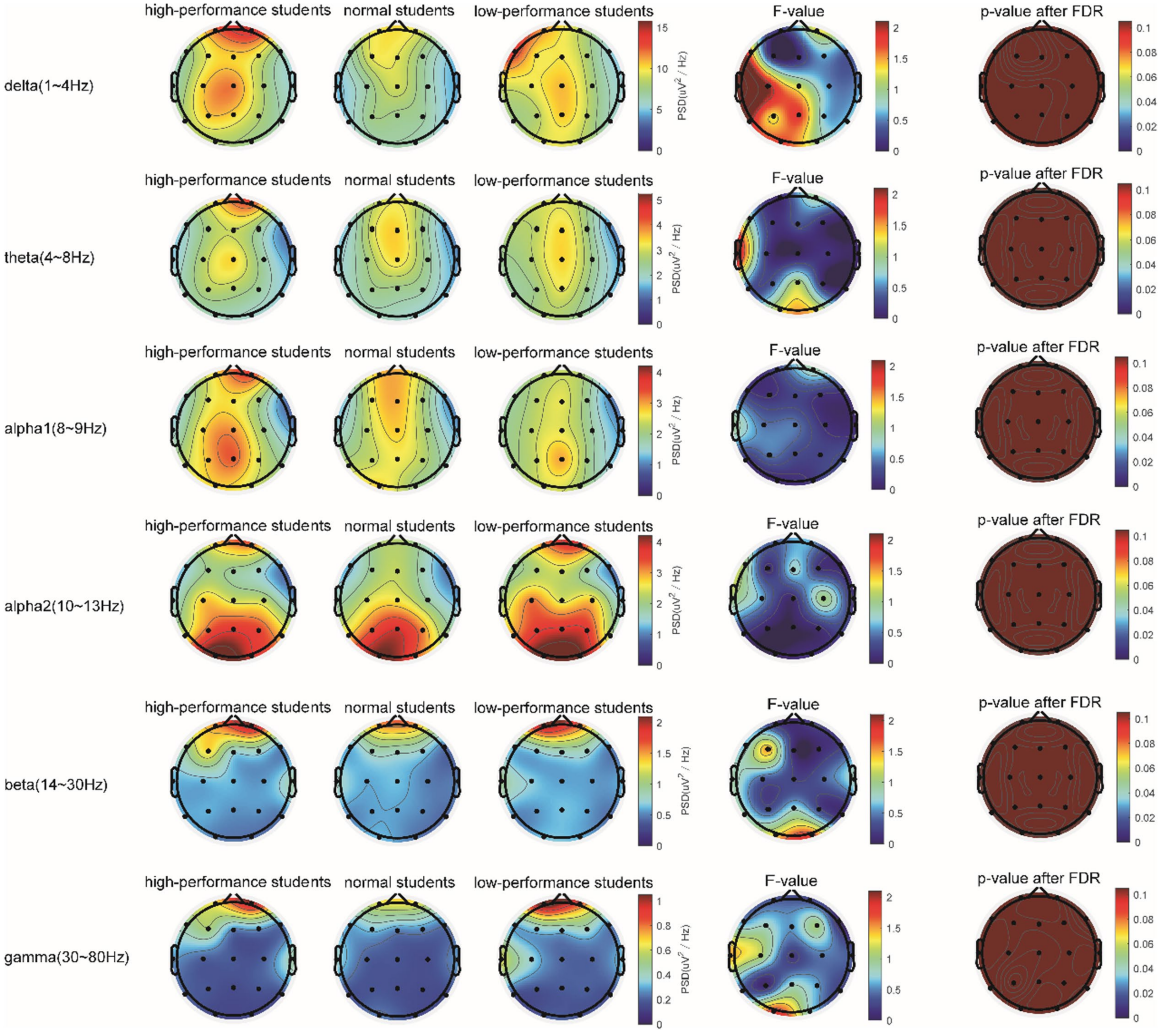
Comparison of the PSD in the δ, θ, α1, α2, β, and γ bands of the three groups of subjects evaluated in the resting state.

In the 5-min resting-state experiment, the spectrum analysis of the three groups of subjects is depicted in Figure 5. No significant differences were observed in the PSD of the three subject groups in the delta, theta, alpha1, alpha2, beta, and gamma bands after the FDR correction. The average PSDs of all 19 electrodes in six frequency bands were extracted to gain a deeper understanding of the overall PSD. An analysis of variance (ANOVA) was conducted on the three subject groups, and no significant differences were identified among them, as shown in Table 3. Consequently, H1a was not supported. No notable differences were observed in whole-brain spectral power between high- and low-performance students concerning resting EEG.

**Table 3:** Analysis of variance for whole-brain PSD (averaged over 19 channels) among the three subject groups in the resting state.

	df	F	Sig.
Delta	2	0.91	0.41
Theta	2	0.19	0.83
Alpha1	2	0.19	0.83
Alpha2	2	0.15	0.86
Beta	2	0.17	0.84
Gamma	2	0.44	0.64

### 4.3. STB experimental score (working memory ability) and frequency domain analysis

The scores from the STB experiments were imported from E-prime 3.0 to SPSS 27 for analysis. In the STB working memory task, one-way ANOVA was employed to assess potential differences in “memory accuracy” and “reaction time” among the three subject groups. The box plot revealed no abnormal values in the data. Levene’s test for homogeneity of variances confirmed that each group’s variance was homogeneous (*p*_correct-rate_ = 0.50; *p*_reaction-time_ = 0.41), The Shapiro–Wilk test indicated that each group’s data did not conform to a normal distribution (*p* < 0.05). However, the one-way ANOVA is relatively stable when deviating from the normal distribution, particularly when each group’s sample size is approximately equal. The non-normal distribution did not significantly impact the probability of committing type I errors, allowing for direct testing. The results demonstrated no statistically significant differences between the three subject groups in terms of correct rate and reaction time (*F*_correct-rate_ = 1.38, *p* > 0.05; *F*_reaction-time_ = 1.85, *p* > 0.05). These findings indicated no significant differences in working memory among the three subject groups (Table 4), suggesting that H2a was not supported. No notable differences were observed in working memory ability between high- and low-performance students.

**Table 4:** Descriptive statistics and ANOVA results of the STB experimental results.

Descriptives								
		N	Mean	Std. deviation	95% confidence interval for mean		Min	Max
					Lower bound	Upper bound		
Correct-rate (%)	High-performance	20	0.88	0.09	0.82	0.93	0.71	1.00
	Normal students	21	0.82	0.11	0.74	0.89	0.63	0.96
	Low-performance	20	0.81	0.12	0.74	0.88	0.50	0.92
Reaction-time(ms)	High-performance	20	1471.68	714.70	1039.79	1903.57	874.92	3328.79
	Normal students	21	1469.18	508.08	1146.36	1792.00	998.92	2724.92
	Low-performance	20	1122.63	261.89	964.37	1280.89	694.21	1513.00

ANOVA						
		Sum of squares	df	Mean square	F	Sig.
Correct-rate (%)	Between groups	0.03	2.00	0.02	1.38	0.27
	Within groups	0.41	58.00	0.01		
Reaction-time(ms)	Between groups	1034911.35	2.00	517455.68	1.85	0.17
	Within groups	9792249.65	58.00	279778.56		

**Figure 6:**
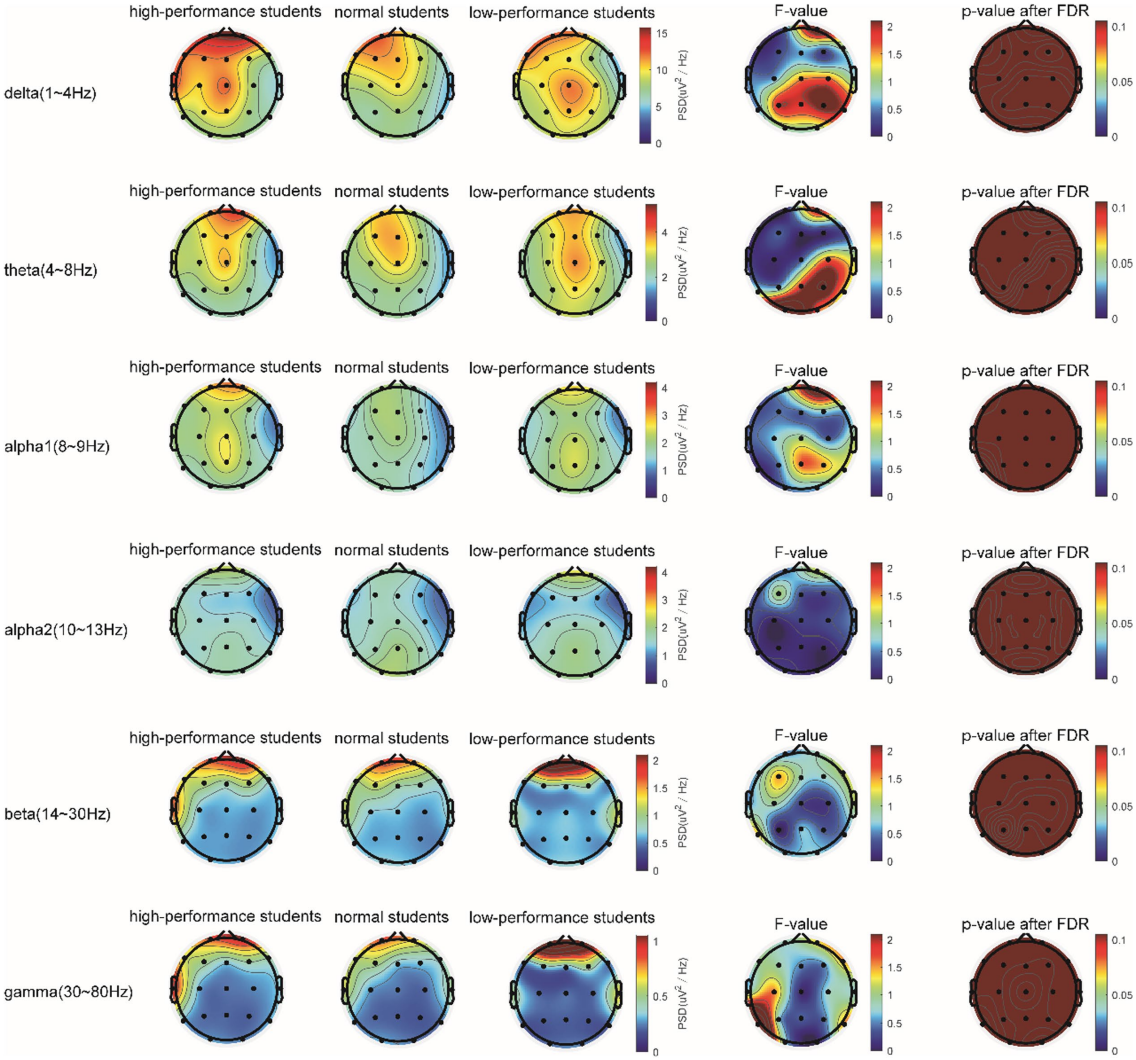
The comparison of PSD in the δ, θ, α1, α2, β, and γ bands of the three groups of subjects in the STB test.

In the STB working memory task, the spectrum analysis of the three subject groups is displayed in Figure 6. No significant differences were detected in the PSD of the three subject groups in the delta, theta, alpha1, alpha2, beta, and gamma frequency bands following FDR correction (Figure 6). The average PSDs of 19 electrodes in six frequency bands were extracted. The results of the variance analysis for the three groups are presented in Table 5. No significant differences were found among the three groups across all frequency bands. Consequently, H2a was not corroborated. No differences were observed in the whole-brain power spectrum between high- and low-performance students concerning working memory tasks.

**Table 5:** Analysis of variance for whole-brain PSD (averaged over 19 channels) among the three subject groups during the STB task.

	df	F	Sig.
Delta	2	0.74	0.48
Theta	2	0.37	0.69
Alpha1	2	1.28	0.29
Alpha2	2	0	0.99
Beta	2	0.1	0.91
Gamma	2	0.2	0.82

### 4.4. Raven test results and frequency domain analysis

A one-way ANOVA was employed to determine if there were any differences in the IQ of the three subject groups, using the results from the Raven test. The Shapiro–Wilk test revealed that the data for each group followed a normal distribution (*p* > 0.05). Levene’s test for homogeneity of variances indicated that the data variance for each group was homogeneous (*p* = 0.88). The ANOVA results showed no significant differences in IQ among high-, low-, and average-performance students (*F* = 0.48, *p* > 0.05; Table 6). Consequently, hypothesis H3a was supported.

**Table 6:** Descriptive statistics and ANOVA results of the Raven test IQ score.

Descriptives							
IQ							
	N	Mean	Std. deviation	95% confidence interval for mean		Min	Max
				Lower bound	Upper bound		
High-performance	20	113.46	3.89	111.11	115.81	107.00	122.00
Normal students	21	113.42	3.15	111.42	115.42	110.00	119.00
Low-performance	20	112.15	4.32	109.54	114.76	105.00	122.00

ANOVA					
IQ					
	Sum of squares	df	Mean square	F	Sig.
Between Groups	14.16	2.00	7.08	0.48	0.62
Within Groups	513.84	58.00	14.68		
Total	528.00	60.00			

**Figure 7:**
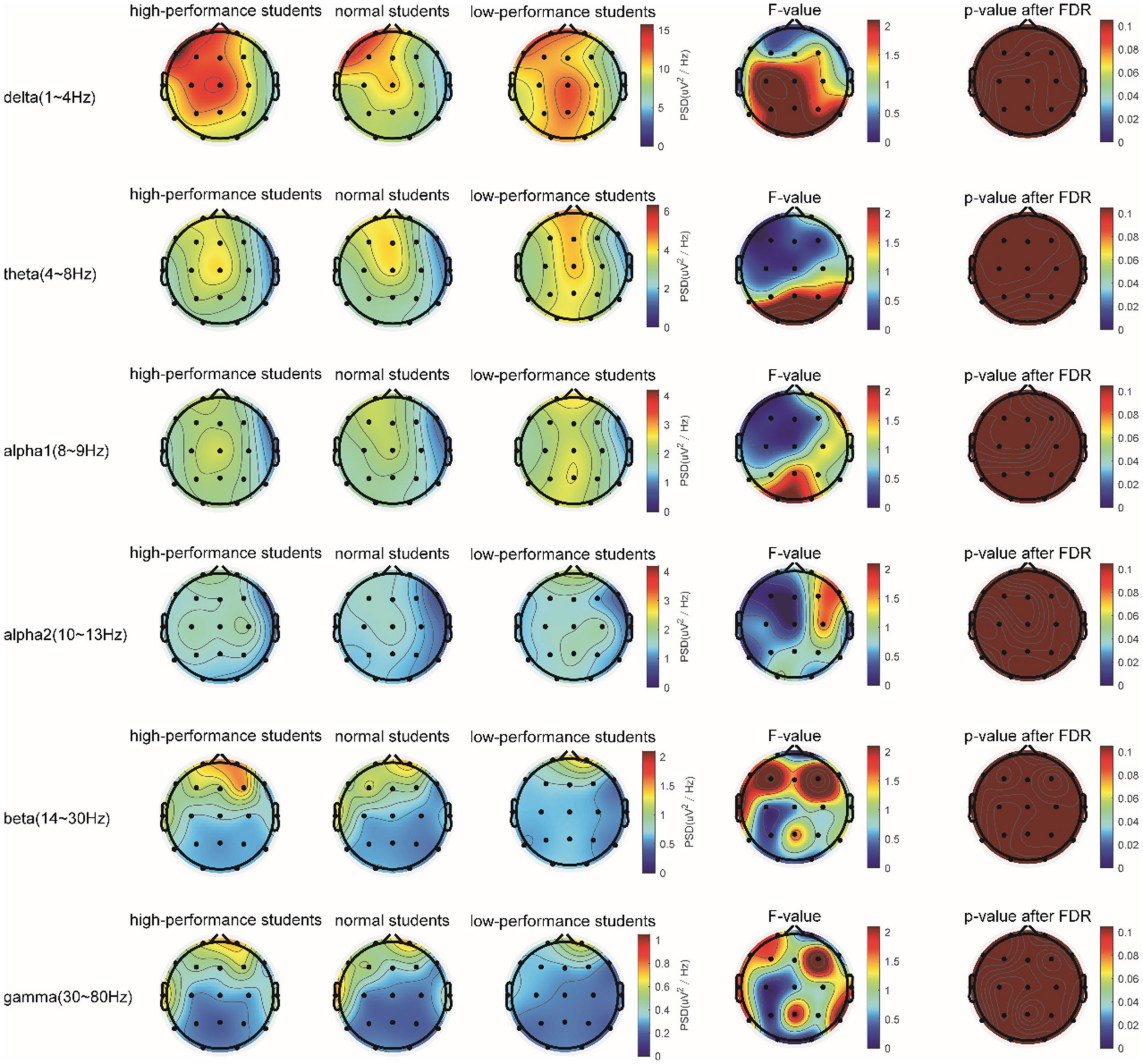
The comparison of the PSD of power spectral density in the δ, θ, α1, α2, β, and γ bands among the three subject groups in the Raven test.

In the Raven test task, the spectrum analysis of the three subject groups is presented in Figure 7. No significant differences were observed in the PSD of the three subject groups for the delta, theta, alpha1, alpha2, beta, and gamma frequency bands following FDR correction (Figure 7). The average PSDs of 19 electrodes in six frequency bands were extracted, and the ANOVA results for the three subject groups are displayed in Table 7. No significant differences were found among the three groups across all frequency bands. As a result, hypothesis H3b was not supported. No significant differences were detected in the whole-brain power spectrum between high- and low-performance students concerning the comprehensive brain ability task.

**Table 7:** Analysis of variance for whole-brain PSD (averaged over 19 channels) among the three subject groups during the Raven task.

	df	F	Sig.
Delta	2	1.40	0.26
Theta	2	1.17	0.32
Alpha1	2	0.89	0.42
Alpha2	2	0.49	0.62
Beta	2	1.42	0.25
Gamma	2	1.41	0.25

### 4.5. Functional connectivity analysis results

**Figure 8:**
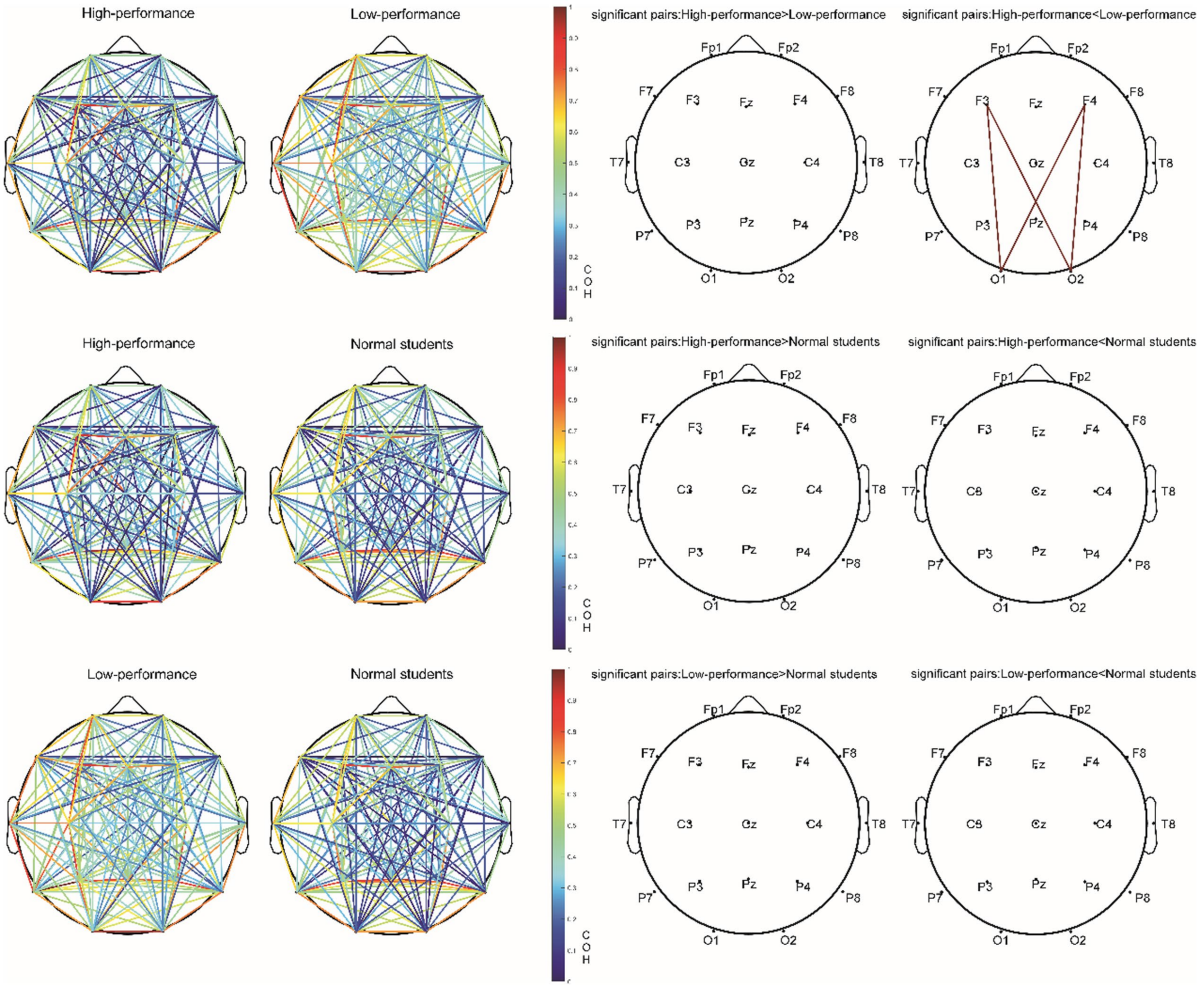
The COH value of the alpha1 (8–9 Hz) band and electrode pair with significant difference (corrected by FDR) in the Raven test experiment.

Following FDR multiple comparisons and corrections, a significant difference was observed solely in the functional connectivity between high- and low-performance students in the alpha1 bands during the Raven test. Students with lower performance exhibited elevated coherence (COH) values within the alpha1 frequency range (8–9 Hz) when compared to their high-performing counterparts. This was particularly evident in the functional connectivity between frontal and occipital regions, as evidenced by the channels F3-O2, F3-O1, F4-O1, and F4-O2. Consequently, hypothesis H3c was supported (Figure 8; Table 8). In the resting state and STB task, no differences were detected in the functional connectivity across all frequency bands (e.g., delta, theta, alpha2, beta, and gamma) among the three student groups (*p* > 0.05). As a result, hypotheses H1b and H2c were not supported.

**Table 8:** T-test results of the COH values of the four electrode pairs with significant differences in the alpha1 frequency band between high- and low-performance students in the Raven test experiment (corrected by FDR).

Electrode pairs	df	T-value	Sig.
F4-O1	38	−4.03	1.31E-04
F3-O1	38	−3.46	6.83E-04
F4-O2	38	−4.32	5.36E-05
F3-O2	38	−3.47	6.63E-04

## 5. Discussion

In this study, we conducted three experiments: resting state, working memory task (STB test), and brain comprehensive ability task (Raven test) to investigate the characteristics of neural oscillation and functional connectivity among three college student groups (high-, average-, and low-performance students). The experimental results revealed no significant differences in power spectral densities across delta, theta, alpha1, alpha2, beta, and gamma bands among the student groups in all three experiments. Additionally, in the STB working memory experiment, no significant differences were found among the three student groups concerning the correct rate, reaction time, and power spectral density of each frequency band. Consequently, it can be concluded that the working memory of low-performance students was not impaired compared to that of high- and average-performance students, and the working memory ability of high-performance students was not significantly superior. Furthermore, no significant differences were observed in IQ scores or power spectral density among the three student groups in the Raven test, indicating no significant differences in IQ or working memory among the students.

Previous studies have demonstrated the effectiveness of working memory training for individuals with cognitive or attention impairments, resulting in substantial gains in working memory performance (Dahlin, 2011). However, research suggests that the effects of such training on healthy individuals are more modest, with only small improvements observed (Loosli et al., 2012; Karbach et al., 2015). According to the compensation theory, high-performing individuals may benefit less from cognitive interventions because they are already functioning at an optimal level, leaving less room for improvement (Titz and Karbach, 2014). It is important to note that our participants, including high-, average-, and low-performing groups, all scored above a very high threshold on the college entrance examination and were admitted to the same university and major. Our university is a highly selective institution with a very high admission threshold, and if the low-performing group had cognitive deficits, they would not have been able to gain admission to the same university and major as the high-performing group. As such, although the high-performing group may have improved their working memory abilities through 3 years of rigorous academic study at the university, this improvement would be minimal. This explains why we did not find a significant difference in working memory performance among high-, average-, and low-performing students in the same university and major.

Spectrum analysis reflects the characteristics of local brain regions, while functional connections indicate the interaction between different regions. In terms of functional connectivity, this study found a significant difference between high- and low-performance students during the Raven test, which was concentrated on the functional connections of the alpha1 bands. Previous studies have demonstrated that in ordinary individuals, rapid formation and dissolution of functional connections can be observed through the synchrony and asynchrony between different brain regions (Herrera-Díaz et al., 2016). In other words, for normal brain functioning, both synchrony and asynchrony of brain regions are required (Friston, 2000; Stam and de Bruin, 2004). The dynamics of functional brain connections can be impaired in two ways: neuronal over-connection or over-disconnection (Stam and van Straaten, 2012).

During the Raven IQ test, low-performing students demonstrated increased coherence (COH) values in the alpha1 band (8–9 Hz) relative to high-performing students, specifically in the functional connectivity between the frontal and occipital regions (F3-O2, F3-O1, F4-O1, F4-O2). The prefrontal lobe is associated with higher cognitive functions such as executive functions, decision-making, and attention, while the occipital lobe is implicated in visual processing. It is posited that low-performing students may adopt a problem-solving strategy on the Raven IQ test that emphasizes information exchange between the frontal and occipital lobes, resulting in augmented functional connectivity within the alpha1 band (Damoiseaux et al., 2006). The most plausible rationale for this observation is that low-performing students might necessitate greater energy expenditure for concentration, suggesting an increased cognitive effort in problem-solving. Alpha waves, particularly in the alpha1 band (8–9 Hz), are typically enhanced during relaxed, closed-eye, or introspective thinking states. The brain might be more inclined to enter such states when attention is diverted, leading to elevated functional connectivity in the alpha1 band. As a result, increased functional connectivity in the alpha1 band could indicate that low-performing students require additional energy to maintain focus during problem-solving (Klimesch, 1999b). Low-performing students might be more susceptible to attentional distractions while engaged in the task. This diversion could lead to heightened alpha1 band functional connectivity, as these students demand more cognitive effort to sustain focus (Eichele et al., 2008). Attentional distractions can result in diminished synchronization of task-related brain regions. When attention is directed toward a specific task, task-related brain regions exhibit increased synchronization. Attentional distractions may induce a decrease in synchronization and a relative enhancement of functional connectivity within the alpha1 band (Doesburg et al., 2009). Attentional dispersion may prompt the brain to continually alternate between processing external stimuli and internal information. In such cases, the functional connectivity of the alpha1 band could function as a “regulator” among different brain regions, allowing the brain to dynamically transition between processing external stimuli and internal information (Palva and Palva, 2007). Additionally, low IQ and poor working memory could contribute to elevated COH in the alpha1 band within frontal and occipital areas (Kane and Engle, 2002). However, the results from our STB and Raven tests negate this possibility, as no significant disparity was found between IQ and working memory among low- and high-performing students.

Another explanation that may lead to the increased functional connection between alpha1 bands is that low-performance students were more prone to fatigue than high-performance students in the task of the comprehensive ability of the brain. Brain fatigue can also lead to increased functional connections in the alpha1 bands, because the brain needs more effort to stay alert and focus (Tanaka et al., 2012). The power of alpha and theta bands has been proven to be a reliable indicator of fatigue-related nerve changes (Zhang et al., 2016; Hsu and Jung, 2017; Majumder et al., 2019). The latest research in cognitive science explores the interaction between brain regions after performing fatigue tasks. The functional connections of the frontal, central, and parietal lobes are closely related to mental fatigue (Liu et al., 2010). Studies have found that the functional connectivity of post-fatigue tasks is closer to that of pre-fatigue tasks, which indicates that the human brain strengthens coupling when tried to maintain information transmission until the required tasks are completed (Chen J. et al., 2018; Han et al., 2019). Compared with the awakened state, the alpha and theta bands in the sleepy state have a higher phase coherence (Chen J. et al., 2018). Therefore, the results of this study probably reflect that low-performance students have not studied hard enough for a long time and that their brains have not been sufficiently trained. Therefore, they are more prone to fatigue than high-performance students in dealing with tasks that consume their brains. However, the brains of high-performance students have been fully trained after 3 years of hard work in college, and their brain anti-fatigue ability is significantly more potent than that of low-performance students regarding comprehensive brain tasks.

A third potential explanation for the observed phenomenon is anxiety, which might contribute to heightened functional connectivity between the frontal (involved in emotion regulation) and occipital lobes (related to visual processing), resulting in increased activity within the alpha1 frequency band (Etkin and Wager, 2007). Extensive research suggests that college students with poor academic performance are more likely to experience mental health issues, with anxiety and depression being the most prevalent psychological concerns (Andrews and Wilding, 2004; Hysenbegasi et al., 2005; Conley et al., 2015; Liu et al., 2022). Anxiety could lead to compromised autonomic regulation within the brain. In states of heightened anxiety, the brain may struggle to effectively regulate its activity, leading to elevated activity in the alpha1 frequency band (8–9 Hz). This could suggest that low-performing students necessitate greater effort in problem-solving due to their brains’ inability to efficiently self-regulate and adapt to task demands (Miskovic and Schmidt, 2010). Anxiety may also give rise to distraction. During states of elevated anxiety, individuals may be more susceptible to distractions from external stimuli and internal thoughts, which could contribute to increased activity in the alpha1 frequency band as the brain demands more effort to maintain attentional focus (Bishop, 2009). Lastly, anxiety might induce hyperactivity between the frontal and occipital lobes. In anxious states, functional connectivity between the frontal (involved in emotion regulation) and occipital lobes (related to visual processing) may be amplified. This enhanced functional connectivity could result in increased activity in the alpha1 frequency band, indicating that the brain requires additional effort to process the task at hand (Etkin and Wager, 2007).

## 6. Limitations

Although our analysis of high- and low-performance students based on study time and absences suggests that the latter group may exhibit reduced self-discipline and indulgence, this conclusion is indirectly inferred. The inclusion of a self-report questionnaire in the study could have provided more direct evidence, albeit with the limitation that students might not be truthful in their responses, particularly regarding sensitive topics such as video gaming habits and partying frequency. Furthermore, we did not account for potential factors influencing motivation, including medical history, physical activity, meditation practice, and social interaction, which may have affected the students’ academic performance and should have been considered in our study design. Our sample was limited to participants from a single university, and we lacked information on family income levels. Notably, research has identified anatomical differences between high- and low-income students that correlate with academic achievement test scores (Mackey et al., 2015).

To address these limitations, we propose conducting a three-year longitudinal study. Repeating the experiments with the same cohort of students at two distinct time points during their college experience (upon university entry and in their third year) would enable longitudinal comparisons between high- and low-performance students. This approach could elucidate the differences between trained and untrained brains by comparing data from freshmen and third-year students. While such a research project would be time-consuming, it could be designed in the future to yield more robust evidence.

## 7. Conclusion

In summary, this investigation examined the characteristics of neural oscillations and functional connectivity among high-, average-, and low-performing college students in resting state, working memory task (STB test), and brain comprehensive ability task (Raven test) conditions. The findings revealed no substantial differences in power spectral densities, working memory, or IQ scores across the three student groups.

In light of our experimental findings, it is important to note that Hypotheses H1a, H1b, H2a, H2b, H3a, and H3b were not supported by the data. No significant differences were observed among high-performing, average, and low-performing students in terms of IQ, working memory, and neural metrics such as Power Spectral Density (PSD) across the three experimental conditions.

However, a minor optimization was observed in high-performance students’ brains compared with low-performance students, primarily manifested in their enhanced concentration, increased fatigue resistance, and reduced anxiety during complex cognitive tasks. This difference is evident in the functional connectivity variations between the frontal and occipital regions in the alpha1 frequency band. Hypothesis H3c received substantial empirical support. Notable differences were found between the high-performing and low-performing student groups in functional connectivity during complex cognitive tasks. Specifically, enhanced functional connectivity was observed in the low-performing student group at brain regions F3-O2, F3-O1, F4-O1, and F4-O2.

The insights gleaned from this research enhance our comprehension of the neural foundations of academic performance and may bear implications for devising targeted interventions and strategies to assist students with diverse levels of academic achievement. Future studies should concentrate on further clarifying the underlying mechanisms and factors contributing to the observed differences in functional connectivity and investigating the potential advantages of targeted interventions to bolster cognitive performance among low-performing students.

## Data availability statement

The datasets presented in this study can be found in online repositories. The names of the repository/repositories and accession number(s) can be found at ScienceDB, https://www.scidb.cn/s/F7z6Jr.

## Ethics statement

The studies involving humans were approved by Hubei university Ethics Committee. The studies were conducted in accordance with the local legislation and institutional requirements. The participants provided their written informed consent to participate in this study.

## Author contributions

ZX paper writer, experiment host, data analysis, and operation. PZ, MT, MZ, and YL laboratory assistant. All authors contributed to the article and approved the submitted version.

## Funding

The work reported in this paper was supported by National Natural Science Foundation, China [No. 71872062].

## Conflict of interest

The authors declare that the research was conducted in the absence of any commercial or financial relationships that could be construed as a potential conflict of interest.

## Publisher’s note

All claims expressed in this article are solely those of the authors and do not necessarily represent those of their affiliated organizations, or those of the publisher, the editors and the reviewers. Any product that may be evaluated in this article, or claim that may be made by its manufacturer, is not guaranteed or endorsed by the publisher.
